# Impact of the COVID-19 Outbreak on Disease Spectrum of Pediatric Intensive Care Units

**DOI:** 10.3389/fmed.2022.801255

**Published:** 2022-05-10

**Authors:** Wen Jing Li, Chun Ling Xue, Zhuo Li

**Affiliations:** ^1^Department of Pharmacy, Children's Hospital of Nanjing Medical University, Nanjing, China; ^2^Department of Emergency, Children's Hospital of Nanjing Medical University, Nanjing, China

**Keywords:** coronavirus disease, COVID-19 outbreak, pediatric intensive care unit, PICU admissions, patient demographics, distribution of disease spectrum, result of etiological examination, PICU length of stay

## Abstract

**Purpose:**

We aimed to analyze the changes in the disease spectrum data of a pediatric intensive care unit (PICU) in Nanjing, China, during the COVID-19 outbreak and explore a feasible plan for the treatment of critically ill children.

**Methods:**

This retrospective study used data from our PICU from 1 January 2018 to 31 December 2020. Patient demographics, distribution of disease spectrum, results of etiological examinations, and the PICU length of stay (LOS) were compared during the COVID-19 period (2020) and the previous years (2018 and 2019).

**Results:**

In 2020, the number of PICU admissions was 46.8 and 47.8% lower than that in 2018 and 2019, respectively. There were significant differences in the number of patients in PICU among different age groups, and these differences were mainly found in children aged <4 years and older than 14 years. The percentage of the number of patients in PICU with respiratory diseases decreased significantly, while those with hematological diseases, poisoning, and rare diseases increased significantly. Moreover, the number of patients with rare diseases increased significantly, while the number of patients with mitochondrial diseases exceeded that of those with autoimmune encephalitis. The PICU LOS in 2020 was higher than that observed in 2018 and 2019, indicating that the changes in the PICU disease spectrum did not directly affect the PICU LOS. Etiological examinations revealed that during the COVID-19 period, the number of patients in PICU with bacterial infections increased, and those with viral infections decreased, although not statistically significant.

**Conclusions:**

A striking decrease in the number of PICU admissions was observed during the COVID-19 outbreak, which caused a significant change in the PICU disease spectrum. Changes in the number and characteristics of patients admitted to PICUs should be considered for facilitating the effective working of PICUs during the COVID-19 pandemic.

## Introduction

Since its onset, COVID-19 has rapidly spread worldwide (1) and has become a global threat (2). COVID-19 spread throughout five continents and the WHO declared the condition a pandemic on 11 March 2020 (3). Our hospital served as the only regional pediatric center in Jiangsu. In response to the COVID-19, our hospital, like many other medical centers, carried out clinical activities while strengthening the pandemic prevention and control measures according to the national unified deployment. However, pediatric intensive care units (PICUs) remained a priority choice for patients with critical diseases and started facing enormous pressure, especially during the early stages of the COVID-19 pandemic.

A similar trend was observed during previous epidemics, such as the Middle East respiratory syndrome (4). Due to the COVID-19 pandemic, there has been a noticeable decrease in the number of emergency department visits in China and other countries worldwide (5), with a similar trend observed for PICU visits (6, 7). As a result, the disease spectrum of PICUs also changed.

The aim of this study was to analyze the changes in the disease spectrum of PICUs before and during the COVID-19 outbreak. It is important to change the diagnostic and therapeutic approaches and ensure an adequate supply of medical resources along with the changes in the disease spectrum in PICU.

## Materials and Methods

### Study Design and Setting

We performed a retrospective observational study in Nanjing, China, from January 2018 to December 2020, in which the data regarding the PICU admissions during the COVID-19 period (1 January 2020 to 31 December 2020) and the previous 2 years (1 January 2018 to 31 December 2018 and 1 January 2019 to 31 December 2019) were compared. The period prior to the COVID-19 outbreak (i.e., during 2018 and 2019) was defined as the reference period since the severe acute respiratory syndrome coronavirus 2 was first isolated from a patient on 7 January 2020, in China, following which the number of confirmed patients with COVID-19 started increasing dramatically (1).

### Selection of Participants

In this study, all patients aged <18 years who were admitted to the PICU of our hospital between January 2018 and December 2020 were included. Patients with missing demographic data were excluded. Each admission of those patients who were admitted multiple times to the PICU during the study period was considered separate.

### Measurement of Data

We compared the patient demographics, diagnosis, disease spectrum, PICU length of stay (LOS), and etiological results between the COVID-19 and reference periods. Data regarding the patient's age, sex, diagnosis, LOS, and etiological results from the PICU were retrieved from the Hospital Information System database. We classified the patients into five age groups according to the textbook Pediatrics: <1 year, 1–3 years, 4–7 years, 8–14 years, and 15–18 years (8). The patient diagnosis was recorded using the International Classification of Diseases, Tenth Revision. Only the primary diagnosis codes were used for the analysis.

The disease spectrum of the PICU was classified into 17 categories according to the classification criteria by Zhao (9): hematological diseases, cardiovascular diseases, digestive diseases, nervous system diseases, endocrine diseases, immunological diseases, urinary diseases, respiratory diseases, tumors, poisoning, accidents, critical neonatal diseases, critical pediatric diseases, critical surgical diseases, rare diseases, allergic reactions, and infectious diseases. Patients with critical neonatal diseases admitted to the neonatal intensive care unit, as well as those with critical surgical diseases, surgical errors, electrical injuries, and child maltreatment admitted to the surgical intensive care unit, were excluded from the disease spectrum.

As our hospital was among the first pediatric hospitals in Jiangsu province to join the Rare Disease Diagnosis and Treatment Network (10), patients with rare diseases were admitted to the PICU using China's First National List of Rare Diseases (11) and Guidelines for the Diagnosis and Treatment of Rare Diseases (2019) (12).

### Statistical Analyses

The number of yearly PICU admissions, average PICU LOS, distribution of yearly disease spectrum, and etiological results during the study period were analyzed to determine the overall trends. The percent decrease for each category was calculated by dividing the number of PICU admissions during the COVID-19 period (2020) by the number of PICU admissions during the reference period (2018 and 2019). Categorical variables were expressed as frequencies and proportions, while continuous variables were represented as medians and interquartile ranges. The chi-square test was used to compare the proportions between the COVID-19 and reference periods. All statistical analyses were performed using the IBM SPSS Statistics (version 26) software (IBM Corp., Armonk, NY, USA). A two-sided *P* < 0.05 was considered statistically significant.

## Results

### Study Population

Overall, 1,372 patients admitted to the PICU from 2018 to 2020 were included in this study. In 2020, 286 patients were admitted to the PICU, with a 46.8 and 47.8% decline compared to the number of PICU admissions in 2018 and 2019, respectively (*P* < 0.05). The proportion of PICU admissions from ER and Wards was 95.5 and 4.5% in 2020, but this is not significant compared to 2018 and 2019. Table 1 shows the characteristics of the patients admitted to the PICU.

**Table 1:** Characteristics of the patients admitted to the pediatric intensive care unit by year (2018–2020).

**Characteristics**	**Total (%)**	**Number of PICU patients in 2018**	**Number of PICU patients in 2019**	**Number of PICU patients in 2020**	*P*-value^*^
Number of PICU admissions	1,372(100.0)	538(100.0)	548(100.0)	286(100.0)	<0.001
Gender, *n* (%)					0.926
Male	823(60.0)	318(59.1)	335(61.1)	170(59.4)	0.939
Female	549(40.0)	220(40.9)	213(38.9)	116(40.6)	0.897
Age (years), *n* (%)					<0.001
<1 year	547(39.9)	232(43.1)	212(38.7)	103(36.0)	0.383
1–3 years	398(29.0)	157(29.2)	174(31.8)	67(23.4)	0.168
4–7 years	213(15.5)	70(13.0)	83(15.2)	60(21.0)	0.039
8–14 years	205(15.0)	78(14.5)	77(14.1)	50(17.5)	0.504
15–18 years	9(0.7)	1(0.2)	2(0.4)	6(2.1)	0.003
LOS (days), mean (SD)	-	13 ± 11.9	15 ± 36.2	16 ± 17.2	0.318

*Data are presented as number (%).*

* *P-values for the change in the number of patients in PICU from 2018 to 2020.*

*PICU, pediatric intensive care unit; LOS, length of stay; SD, standard deviation*.

In 2020, the decline in the number of PICU-admitted patients classified based on their sex was similar to that observed for PICU admissions. However, these results were not statistically significant between male children and female children (*P* > 0.05). The number of patients admitted to the PICU decreased in the <1 year and 1–3 years age groups and increased in the 4–7, 8–14, and 15–18 years age groups. This increase was statistically significant in the 15–18 years age group. The differences in the number of PICU admissions and patients in PICU classified by age were statistically significant (*P* < 0.001 and *P* = 0.003, respectively).

The PICU LOS in 2020 has increased compared to that in 2018 and 2019 by 23.1 and 6.7%, respectively, although not statistically significant (*P* > 0.05).

### Disease Spectrum

In 2018 and 2019, the top three most common chief diseases were respiratory diseases, nervous system diseases, and critical diseases. However, this changed in 2020, with the top three most common chief diseases being nervous diseases, respiratory diseases, and critical diseases.

Table 2 shows the number of PICU admissions classified by disease or injury. In 2020, the percentage of the number of patients in PICU with respiratory diseases decreased significantly, while those with hematological diseases, poisoning, and rare diseases increased significantly. Patients who were diagnosed with autoimmune encephalitis still accounted for a large proportion. However, the types of rare diseases and the number of patients diagnosed with inherited metabolic disorders increased significantly, with the number of patients with mitochondrial diseases exceeding that of those with autoimmune encephalitis.

**Table 2:** Distribution of the disease spectrum of patients admitted to the pediatric intensive care unit by year (2018–2020).

**Variable, *n* (%)**	**Number of PICU patients in 2018**	**Number of PICU patients in 2019**	**Number of PICU patients in 2020**	*P*-value^*^
Hematological diseases	9(1.7)	10(1.8)	12(4.2)	0.055
Cardiovascular diseases	30(5.6)	28(5.1)	10(3.5)	0.448
Digestive diseases	23(4.3)	20(3.7)	15(5.2)	0.581
Nervous system diseases	119(22.1)	126(23.0)	69(24.1)	0.874
Endocrine diseases	4(0.7)	9(1.6)	1(0.4)	0.157
Immunological diseases	18(3.4)	8(1.5)	10(3.5)	0.099
Urinary diseases	6(1.1)	7(1.3)	3(1.1)	0.950
Respiratory diseases	206(38.3)	225(41.1)	64(22.4)	<0.001
Tumors	9(1.7)	12(2.2)	8(2.8)	0.572
Poisoning	7(1.3)	5(0.9)	15(5.2)	<0.001
Accidents	15(2.8)	16(2.9)	3(1.1)	0.228
Critical diseases	71(13.2)	55(10.0)	41(14.3)	0.198
Rare diseases	8(1.5)	14(2.6)	29(10.1)	<0.001
Allergic reactions	1(0.2)	3(0.6)	0(0.0)	0.322
Infectious diseases	12(2.2)	10(1.8)	6(2.1)	0.896

*Data are presented as number (%).*

* *P-values for the change in the distribution of the disease spectrum of patients in PICU from 2018 to 2020*.

### Etiological Results

Table 3 shows the etiological results of the patients admitted to the PICU from 2018 to 2020. The etiology determined was classified based on the pathogen species as follows: bacteria, virus, fungus, *Mycoplasma*, and *Mycobacterium tuberculosis*. A total of 552 positive results were detected in 2020, with a 47.1 and 48% decline compared to that obtained in 2018 and 2019, respectively (*P* < 0.05).

The number of pathogen species detected remained similar for the COVID-19 and reference periods. The number of positive results for each pathogen detected were decreased compared to that in 2018 and 2019, however, the percentage of patients in PICU with positive results for bacterial detection increased, while those with positive results for virus detection decreased, although not statistically significant (*P* > 0.05).

**Table 3:** Etiological results of the patients admitted to the pediatric intensive care unit by year (2018–2020).

**Pathogen species**	**Number of PICU patients in 2018**,		**Number of PICU patients in 2019**,		**Number of PICU patients in 2020**,		*P*-value^*^
	***n* (%)**	***N***	***n* (%)**	***N***	***n* (%)**	***N***	
Bacteria	371(35.5)	39	436(37.6)	39	241(43.7)	37	0.105
Virus	452(43.3)	10	461(39.7)	8	199(36.1)	8	0.173
Fungus	29(2.8)	6	38(3.3)	8	13(2.3)	5	0.565
*Mycoplasma*	192(18.4)	1	226(19.4)	1	99(17.9)	1	0.782
Total	1,044(100.0)	56	1,161(100.0)	56	552(100.0)	51	<0.001

*Data are presented as number (%).*

* *P-values for the change in the etiological results of the patients from 2018 to 2020.*

*N, total number of the pathogen species*.

## Discussion

In this retrospective study, a significant decrease was observed in the number of PICU admissions during the COVID-19 outbreak compared to that in 2018 and 2019, which could be theoretically expected due to the strict home quarantine order (13). Previous studies on decreases in pediatric admissions or visits due to the COVID-19 outbreak have mainly focused on the emergency departments in some areas (14) or several countries (15–19). Although significant attention has been paid to PICU admissions with great differences in some areas, little attention has been paid to the impact of the COVID-19 outbreak on the disease spectrum of PICUs (20, 21). Thus, our study was among the first to analyze the impact of the COVID-19 outbreak on the disease spectrum of PICUs. In a multicenter study in Brazilian PICUs during the period of physical distancing and school closing (7), there was a drop in bronchiolitis, asthma, and community-acquired pneumonia admissions, but no effect on hospitalization rates in epilepsy, diarrhea, sepsis, bacterial meningitis, or surgery. Our study was among the first to analyze the impact of the COVID-19 outbreak on the disease spectrum of PICU in China, and different conclusions were obtained by comparing the data over 3 years.

In our study, the number of PICU admissions during the COVID-19 period declined by 46.8 and 47.8% compared to that in 2018 and 2019, respectively. The number of pediatric hospitalizations admissions decreased by 13.7 and 18.9%, compared to that in 2018 and 2019, respectively.

However, the decrease in the number of PICU admissions was more than that observed in pediatric hospitalizations admissions. The decrease in PICU admissions may be explained by several factors. First, fear of being exposed to COVID-19 may have had an impact on the health-seeking behavior of people who thought hospitals were the primary locations of virus transmission. Second, travel was suspended from multiple cities, social activities were canceled, outdoor activities were avoided, and other social distancing measures may have resulted in a decreased transmission of infectious diseases and leading to a decrease in the incidence of some diseases. Third, in order to respond to the epidemic, most hospitals closed routine clinics and canceled elective surgeries, it became difficult to seek medical care in areas with scarce medical resources and delayed admissions and sicker patient admission should be taken into account. As such, understanding the changes in the disease spectrum of PICUs during the COVID-19 pandemic is important for ensuring an adequate supply of medical resources.

Our study results indicated that the number of patients in PICU in 2020 decreased in the <1 year and 1–3 years age groups, which could have been possible as outdoor activities were avoided during the COVID-19 pandemic (22, 23). Conversely, the number of PICU admissions significantly increased in the 15–18 years age group who were diagnosed with nervous system diseases, and rare diseases increased compared to 2018 and 2019 which is consistent with the changes in the disease spectrum. However, this increase was not associated with schools closing during COVID-19. It is to be considered that the safety and mental health of adolescents should be concerned when schools were closed during COVID-19 according to reports in the literature (24, 25), although there were no cases of accidental poisoning or deliberate self-harm in our study.

Additionally, the disease spectrum of PICUs significantly changed during the COVID-19 period. Respiratory diseases such as upper and lower respiratory infections were no longer the most common chief disease of PICUs due to the reduction of cross-infection, a reduction in environmental pollution (26, 27), and the use of personal protective equipment during the COVID-19 period (28). A similar decrease in the incidence of patients in PICU with outdoor accidents was also observed; in contrast, drowning continued to be the major source of outdoor accidents despite the extension of staying at home. The incidence of patients in PICU with drug or pesticide poisoning increased significantly during social distancing periods, although outdoor activities were limited. Thus, the reasonable placement and management of common medicines and pesticides to prevent drug and pesticide poisoning in children (29), as well as the effective care of children, especially young children (30), remains questionable. Since patients with critical diseases could not be easily treated in primary medical institutions, the change in the number of patients in PICU with critical diseases during the COVID-19 outbreak was relatively small. Hence, PICUs in tertiary hospitals should be prepared to accept patients transferred from other hospitals during a pandemic.

The most significant change observed in the disease spectrum of the PICU was an increase in the number and types of rare diseases, such as inherited metabolic disorders, autoimmune encephalitis, spinal muscular atrophy, and urea cycle disorders (11). In our study, the number of patients in PICU with inherited metabolic disorders, especially mitochondrial diseases, exceeded that of those with autoimmune encephalitis during the COVID-19 outbreak, indicating that the original disease spectrum of PICUs, which mainly includes respiratory infectious diseases, has changed, and a variety of rare diseases should be taken into account. It is imperative to establish a rare disease database by accumulating relevant diagnoses and treatment experiences (31).

The social distancing measures during the COVID-19 pandemic could have resulted in a decrease in close contact between people, leading to a decreased transmission of infectious or self-limited diseases caused by the other types of viruses (28, 32), corresponding to a decrease in the percentage of positive results for virus detection. The increase in the percentage of positive results for bacterial detection may be explained by the following factors. First, the proportion of respiratory diseases caused by viruses decreased and that caused by bacteria increased. Second, some diseases such as nervous system diseases, hematological diseases, and rare diseases, were prone to nosocomial infection caused by bacteria because of the complexity and severity. The etiological results suggested that we should focus on the control of nosocomial infection caused by bacteria in PICU while preventing the spread of COVID-19.

A comparison of the average LOS in the PICU from 2018 to 2020 showed that the LOS in 2020 was longer than that in 2018 and 2019 and was associated with an extended LOS of some critical patients in PICU, according to data from the original medical records. These findings indicated that the changes in the disease spectrum of the PICU did not directly affect the LOS, which was consistent with the fact that the severity of the illness plays a decisive role in the LOS (33).

This study had several important limitations. First, this study was conducted at the PICU of a tertiary hospital in Nanjing, China; thus, our results may provide limited knowledge to other PICUs. Nonetheless, our PICU, which is the core PICU in the Jiangsu and Anhui provinces, provided care to a large portion of pediatric patients in the region. Second, this was a retrospective observational study, and we may not have captured all the data required to explain the changes in the PICU admissions during the COVID-19 period. There may have been unrecognized factors other than COVID-19 that may have affected the results of our study. For example, the use of video-conferencing for online handovers (34, 35) and multidisciplinary hybrid virtual and physical clinical rounds (36) in the PICU were perceived as feasible to maintain social distancing between team members during the COVID-19 pandemic, thereby reducing nosocomial infection, which was not adopted in our PICU, because we did not admit patients with COVID-19, and infectious diseases account for a small part in our unit. Third, the data capture of this study covered the entire year of 2020, and the patterns in PICU admissions during the early phase of the COVID-19 pandemic may be different from those in the later stage. Future studies can be performed using longer study periods to observe the changes in PICU admissions during the advent of the post-COVID-19 era.

## Conclusion

In this study, we analyzed the changes in the disease spectrum of a PICU before and during the COVID-19 outbreak. A striking decrease in the number of PICU admissions was observed, which was associated with the home quarantine policy and the measures of wearing masks due to the COVID-19 pandemic. Changes in the number and characteristics of patients admitted to PICUs should be considered in the rational allocation of PICU resources during the COVID-19 pandemic. Although we did not observe any critical cases due to delayed hospital admission in our study, the risk of avoidance or delay of admissions to critically ill children may only become apparent in the months and years to come. Future studies that include data from longer study periods are needed to further explore the impact of these changes during the advent of the post-COVID-19 era.

## Data Availability Statement

The original contributions presented in the study are included in the article/supplementary material, further inquiries can be directed to the corresponding author.

## Ethics Statement

Ethical review and approval was not required for the study on human participants in accordance with the local legislation and institutional requirements. The requirement for informed consent was waived owing to the retrospective nature of the study.

## Author Contributions

WL and ZL contributed to the conception and design of the study. WL and CX organized the database and performed the statistical analysis. WL wrote the first draft of the manuscript. ZL and CX wrote sections of the manuscript. All authors contributed to manuscript revision, read, and approved the submitted version.

## Funding

All phases of this study were supported by the Medical Science and Technology Development Foundation, Nanjing Municipality Health Bureau (2020, YKK 20130), and Jiangsu Hospitals Association (JSYGY-3-2021-JZ66).

## Conflict of Interest

The authors declare that the research was conducted in the absence of any commercial or financial relationships that could be construed as a potential conflict of interest.

## Publisher's Note

All claims expressed in this article are solely those of the authors and do not necessarily represent those of their affiliated organizations, or those of the publisher, the editors and the reviewers. Any product that may be evaluated in this article, or claim that may be made by its manufacturer, is not guaranteed or endorsed by the publisher.

## Abbreviations

COVID-19: coronavirus disease
LOS: length of stay
PICU: pediatric intensive care unit.
